# The Rise of Scalable Micro/Nanopatterning

**DOI:** 10.3390/mi8090275

**Published:** 2017-09-11

**Authors:** Ke Du, Ishan Wathuthanthri, Chang-Hwan Choi

**Affiliations:** 1 Department of Chemistry, University of California, Berkeley, CA 94720, USA; kdu1@berkeley.edu; 2 Northrop Grumman Mission Systems, Advanced Technology Labs, Linthicum, MD 21090, USA; Ishan.Wathuthanthri@ngc.com; 3 Department of Mechanical Engineering, Stevens Institute of Technology, Hoboken, NJ 07030, USA

This is the golden age of scalable micro/nanopatterning, as these methods emerge as an answer to produce industrial-scale nano-objects with a focus on economical sustainability and reliability. The improvement of scalability and reliability in nanomanufacturing is a key step to move nanotechnology advances closer to the customer market. This special issue in *Micromachines* aims to present the most recent research developments in scalable micro/nanopatterning. A total of eight papers are presented, including three review papers and five original research papers. The topics include “top-down” approaches, “bottom-up” approaches, and the combination of “top-down” and “bottom-up” approaches.

The Scalable Nanomanufacturing (SNM) program of the National Science Foundation (NSF) was initiated in 2011, and provides funding support to research and education to accelerate the commercialization of nanoscale inventions. Khershed Cooper (NSF) reviewed the initiation of the SNM program [1]. The importance of and the need for scalable nanomanufacturing were outlined. The applications and recent advances in scalable nanomanufacturing were also discussed. More importantly, challenges for nanomanufacturing were listed, which point out the future research directions that can enable high-value end applications.

Hu et al. provided a review of tip-based nanofabrication (TBN) for scalable manufacturing [2]. TBN techniques use a nanometer scale tip to pattern nanostructures. The history of TBN was reviewed, followed by the introduction of various TBN techniques. The focus was put on those TBN techniques that have demonstrated potential for scalable nanomanufacturing.

Du et al. provided a review of stencil lithography, which is a scalable and resistless nanomanufacturing technique [3]. The current state-of-the-art of stencil lithography was reviewed followed by a discussion of the challenges and outlook of stencil lithography.

Chauvin et al. reported a simple and scalable fabrication technique of porous gold nanowires based on laser interference lithography and dealloying [4]. The gold-silver alloy nanowires were created by using “lift-off” followed by an annealing process. Then, nanoporous gold nanowires were formed by a dealloying process using concentrated nitric acid. The introduced technique provides a rapid and low-cost method to fabricate complicated quasi-3D metallic nanostructures.

Quantum-dots (QD) are semiconductor particles and have been widely used in the fields of biomedical sensing, photovoltaic devices, and nano-electronics. Keum et al. introduced a scalable QD patterning technique using shape memory polymer (SMP) [5]. The adhesion of the SMP stamp to the QD coating was controlled by simply changing the temperature below and above the glass transition temperature of SMP. This enables scalable, rate-independent, and well-ordered QD patterning for future applications such as next-generation photovoltaic devices and transistors.

Nanoimprint lithography (NIL) is a rapid and high resolution nanomanufacturing technique. Lin et al. reported a scalable, time-efficient, and high resolution fabrication process of silicon oxide [6]. An NIL mold was fabricated by using electron-beam lithography (EBL) followed by deep reactive ion etching on a silicon substrate. After that, it was used to imprint on a resist-coated silicon oxide substrate. Large-area silicon oxide nanostructures were formed by reactive ion etching and resist stripping. Unlike traditional multi-spot exposure in EBL, a one-spot exposure was applied and improved the time efficiency by more than 4000 times, which significantly improved the patterning speed.

Hoshian et al. introduced a non-lithographic method to pattern silicon [7]. Inkjet printing was used to define the patterning features and metal-assisted chemical etching (MaCE) was used to etch the silicon substrate. This etching process resulted in silicon nanowires in the patterning trench, which is a simple and efficient way to pattern hierarchical nanostructures. Such nanowires can be removed from the trench by performing potassium hydroxide etching. The whole fabrication process does not require vacuum-based lithography and a dry etching process, which significantly reduces the patterning costs.

Ou et al. demonstrated antireflective SiC nanospikes with a simple and low-cost process by using metal nanoparticles as an etching mask [8]. The metal nanoparticles were formed by a rapid thermal annealing of a thin metal film. Tuning the metal film thickness changed the dimensions of the SiC nanospikes and also changed the transmission/absorption spectrum. This scalable nanopatterning technique is a promising method to fabricate optoelectronic devices.

The future of scalable micro/nanopatterning is bright. We hope that this special issue will help scientists and engineers to develop more advanced micro/nanopatterning techniques in the near future. We would like to thank all the contributors to this special issue and the reviewers for providing valuable comments to the submitted manuscripts.
